# Incidence and progression of diabetic retinopathy in Sub-Saharan Africa: A five year cohort study

**DOI:** 10.1371/journal.pone.0181359

**Published:** 2017-08-02

**Authors:** Philip I. Burgess, Simon P. Harding, Marta García-Fiñana, Nicholas A. V. Beare, Simon Glover, Danielle B. Cohen, Gerald Msukwa, Theresa J. Allain

**Affiliations:** 1 Malawi-Liverpool-Wellcome Trust Clinical Research Programme, Queen Elizabeth Central Hospital, Blantyre, Malawi; 2 Department of Eye and Vision Science, University of Liverpool, Liverpool, United Kingdom; 3 Department of Biostatistics, University of Liverpool, Liverpool, United Kingdom; 4 Lions Sight First Eye Unit, Queen Elizabeth Central Hospital, Blantyre, Malawi; 5 College of Medicine, University of Malawi, Blantyre, Malawi; Soochow University Medical College, CHINA

## Abstract

**Aims:**

To describe the incidence and progression of retinopathy in people with diabetes in Southern Malawi over 5 years. To document visual loss in a setting where laser treatment is not available.

**Methods:**

Subjects from a cohort sampled from a hospital-based, primary-care diabetes clinic in 2007 were traced in 2012. Laser treatment was not available. Modified Wisconsin grading of retinopathy was performed using slit lamp biomicroscopy by a single ophthalmologist in 2007 and using four-field mydriatic fundus photographs at an accredited reading centre in 2012. Visual acuity was measured by Snellen chart in 2007 and by ‘Early Treatment of Diabetic Retinopathy Study’ chart in 2012. HbA1c, blood pressure, HIV status, urine albumin–creatinine ratio, haemoglobin and lipids were measured.

**Results:**

Of 281 subjects recruited in 2007, 135 (48%) were traced and assessed, 15 were confirmed dead. At follow-up (median 5.3 years) ≥2 step retinopathy progression was observed in 48 subjects (36.4%; 95% CI 28.2–44.6). Incidence of sight threatening diabetic retinopathy for those with level 10 (no retinopathy) and level 20 (background) retinopathy at baseline, was 19.4% (11.3–27.4) and 81.3% (62.1–100), respectively. In multivariate analysis 2 step progression was associated with HbA1c (OR 1.2495%CI 1.04–1.48), and haemoglobin level (0.77, 0.62–0.98). 25 subjects (18.8%) lost ≥5 letters, 7 (5.3%) lost ≥15 letters.

**Conclusions:**

Progression to sight threatening diabetic retinopathy from no retinopathy and background retinopathy was approximately 5 and 3 times that reported in recent European studies, respectively. Incidence of visual loss was high in a location where treatment was not available.

## Introduction

Sub-Saharan Africa faces an epidemic of diabetes [1]. In contrast to high income countries [2–8] very few cohort studies have investigated prevalence, incidence and determinants of severity and progression of diabetic retinopathy (DR) in this region [9]. Little is known about the effects of infectious disease (including Human Immunodeficiency Virus (HIV) and malaria) and anemia on the microvascular complications of diabetes. Malawi has a population of 16.4 million. Annual per capita healthcare expenditure is extremely low at US$77 [10]. The best available population based survey (World Health Organisation (WHO) STEPwise methods) reported a prevalence of diabetes of 5.6% in Malawian adults in 2009 [11]. In 2007, a cross sectional study of diabetes complications was performed at the diabetes clinic at Queen Elizabeth Central Hospital (QECH), Blantyre [12]. As part of this study our group performed slit lamp bio-microscopy to document grades of retinopathy. We found a high prevalence of both sight-threatening and proliferative DR: 19.6 and 5.7%, respectively [13]. At the time of this survey, and until 5 years later, laser treatment was not available in the public sector in Blantyre. In 2012 we recalled subjects from this cross sectional study in order to report retinopathy progression at 5 years. Additionally, we undertook a prospective, 2 year cohort study of patients attending two hospital-based diabetes clinics. Data from this study has been published elsewhere [14,15].

## Materials and methods

### Setting

QECH is a large teaching hospital. QECH provides primary and secondary care to the people of greater Blantyre (approximately 1 million). Tertiary care is provided to the Southern Region of Malawi. At the time of this study (2012) the QECH diabetes clinic was the only public sector diabetes clinic in Blantyre. In the period 2007 to 2012 the clinic underwent a number of changes. The number of registered patients increased from approximately 800 to 2000. A vibrant nurse-led patient education programme supported by the World Diabetes Foundation commenced in 2008. Its aims were improving compliance with diet and medications and educating patients on the complications of diabetes. An electronic records system (Diabetes and Hypertension System, Baobab Health Trust, Malawi) was installed in early 2010. In 2007 medications regularly available free of charge were glibenclamide and insulin (lente and soluble). Metformin was available from private pharmacies but rarely from the hospital pharmacy. By 2012 metformin was more frequently available free of charge. However, supplies of all drugs remained intermittent. Tests available at the clinic were the same in 2007 as 2012: glycaemic control measured by fasting blood glucose (FBG), blood pressure (BP), height and weight. Measurement of lipids, glycosylated haemoglobin (HbA1c) and urine test sticks for microalbuminuria were not available routinely.

### Participants

Patient selection in the 2007 cross sectional study has been described elsewhere [12,13]. Briefly, consecutive subjects attending for routine out-patient review between March and June 2007 were invited to participate. Of 620 subjects included in the study 281 were examined by an ophthalmologist. Sampling was *ad hoc* (i.e. not consecutive): subjects had slit lamp examination if the ophthalmologist was present at the particular clinic at which they were recruited. At this time laser treatment was not available in the public sector. The 2007 study was not planned as a cohort study therefore no contact details were recorded. Tracing of subjects between December 2011 and November 2012 was systematic. The QECH diabetes clinic electronic patient record system was searched by subject name by a research nurse. Identified persons were then contacted by phone or home visit. The study team attended the diabetes clinic weekly between May and November 2012 to approach patients in the clinic waiting room. The majority of deaths in Malawi are not registered. The relatives of deceased subjects were visited at home by a study nurse in order to confirm the death. Death was recorded if confirmed by a first degree relative or ‘Traditional Authority’ (village leader in rural districts).

### Procedures

Clinical assessment in the 2007 study has been described elsewhere [12,13]. Briefly, visual acuity (corrected with pin-hole) was measured using a Snellen chart. FBG, HbA1c and HIV status were tested. Slit lamp biomicroscopic retinopathy grading was performed by one ophthalmologist (SG). Retinopathy and maculopathy were classified by feature-specific grading as described in the Liverpool Diabetic Eye Study (LDES) [16] (S1 Fig).

Clinical assessment of subjects in 2012 was the same as in our 24 month cohort study described in detail elsewhere [14]. Briefly, uncorrected and pinhole visual acuity was assessed using an Early Treatment of Diabetic Retinopathy Study (ETDRS) chart. Thresholds for moderate visual impairment (50 to 59 letters; equivalent to 6/24 Snellen) and severe visual impairment or blindness (<50 letters; equivalent to 6/36 or worse) were set according to the WHO [17]. For subjects with corrected visual acuity in the better eye of less than 80 letters, the principle cause of vision loss was documented by the examining ophthalmologist (PB). Hypertension was defined according to WHO criteria [11]: systolic blood pressure ≥140 mmHg, or diastolic blood pressure ≥ 90 mmHg, or taking anti-hypertensive medication. Point-of-care testing was offered for haemoglobin level and HIV (Malawian national protocol [18]). Anemia was defined according to the WHO: male 130 g/l; female 120 g/l [19]. Laboratory testing of venous blood samples was performed for HbA1c, fasting glucose, HDL cholesterol, LDL cholesterol, triglycerides and serum creatinine. Urine albumin–creatinine ratio was measured.

As in 2007 retinopathy and maculopathy were classified by feature-specific grading using the LDES scale [16]. In contrast to 2007 dual grading of digital fundus photos of four 45° standard fields [16] was performed at the Liverpool Reading Centre by accredited graders. Sight threatening diabetic retinopathy (STDR) was defined as any of the following: retinopathy level 40–71+ (moderate pre-proliferative DR or worse); level 3–4 maculopathy (macular exudates in a circinate pattern or within one disc diameter of the foveal centre or CSME (ETDRS definition [20])); or other retinal vascular disease related to diabetes: central or branch retinal vein occlusion, central or branch retinal artery occlusion.

### Statistical analysis

Retinopathy grades were analysed by patient according to the worse or only gradeable eye. Visual acuity scores were analysed by patient according to the better eye. For the purposes of analysis visual acuity scores from 2007 (corrected Snellen acuities in the better eye) were converted to ETDRS letter scores using a standard conversion table [21]. Comparison was then made with 2012 ETDRS letters measurement in the better eye. Primary outcome was progression of retinopathy by ≥2 steps on the LDES scale (either 2 step progression in one eye *or* 1 step progression in both eyes). A multiple logistic regression model (backwards stepwise; probability of removal of 0.2) was constructed to determine the odds ratio and 95% confidence intervals for the primary end point at 5 years. For the majority of variables the baseline value was used in the analysis. For those variables only measured at the 2012 visit this value was used (uACR, LDL, HDL, triglycerides). An initial 11 variables were studied: HbA1c, duration of diabetes, baseline grade of DR, type of diabetes, sBP, haemoglobin level (2012), urine albumin creatinine ratio (uACR)(2012), triglycerides (2012), HIV status, age and sex. uACR did not demonstrate a linear association with probability of 2 step progression; a logarithmic transformation (base 10) was used in analysis. All tests were two-sided and a p value <0.05 was taken to indicate statistical significance. All calculations were performed using STATA version 12 (StataCorp, College Station, TX, USA). The study was approved by the University of Malawi, College of Medicine Research Ethics Committee and the University of Liverpool Research Ethics Committee. All participants gave written informed consent.

## Results

A total of 135 subjects were assessed between December 2011 and December 2012. Median time to follow up was 5.3 years (IQR 5.1–5.4). 15 subjects were confirmed dead. (Total follow-up 150 subjects: 53% of the original 281 subject cohort) (S2 Fig). Baseline characteristics of 281 subjects included in the 2007 study categorised by follow-up are shown in Table 1. Compared to subjects who were traced and assessed in 2012, subjects who were not seen in 2012 demonstrated higher median age and systolic BP and a higher prevalence of STDR (based on univariate statistical tests with no adjustment for multiple comparisons). Subjects not seen in 2012 demonstrated worse baseline visual acuity scores in the better eye (data not shown; p = 0.0001, Χ^2^ test for trend). Table 2 shows demographic, clinical and biochemical measurements for subjects included in the 2007 study and seen again in 2012 categorised by grade of retinopathy at baseline.

**Table 1:** Baseline (2007) demographic, clinical and biochemical measurements and retinopathy grading for 281 subjects included in the 2007 study categorised by follow-up: Traced and assessed in 2012 (n = 135) or not seen in 2012 (n = 146). Subjects not seen in 2012 further classified into those confirmed dead (n = 15) and those lost to follow-up (n = 131). Statistical comparison (analysis of bias): subjects seen in 2012 (n = 135) vs subjects not seen in 2012 (n = 146).

Characteristic	Subjects not seen in 2012 (n = 146)		Subjects seen in 2012 (n = 135)	p value
	Confirmed dead (n = 15)	Lost to follow-up (n = 131)		
Age (yrs; med, IQR)	60 (57–62)	57 (47–65)	52.0 (45–58)	p = 0.0014*†
Type 1 diabetes	0	14 (10.7%)	18 (13.3%)	p = 0.352 ǂ
Duration (yrs; med, IQR)	8.7 (6.3–13.7)	4.8 (1.7–9.7)	3.9 (2.2–7.8)	p = 0.234 ł
sBP (mmHg; med, IQR)	160 (120–171)	140 (120–157)	130 (120–150)	p = 0.015*†
HbA1c (NGSP%; mean,SD)	9.9 (2.3)	9.3 (2.2)	9.4 (2.6)	p = 0.727 †
HIV positive	1 (6.6%)	14 (10.7%)	15 (11.1%)	p = 0.849 ǂ
Urine dipstick	None 2; trace 3; 1+ 3; 2+ 3; 3+ 2; 4+ 0; No data 2	None 27; trace 39; 1+ 22; 2+ 8; 3+ 4; 4+ 3; No data 28	None 43;trace 44;1+ 29; 2+ 6; 3+ 5; 4+ 1	p = 0.06 ≠
Any DR (n; %; 95% CI)	9 (60%) (35.2–84.8)	39 (29.8%) (22.0–37.6)	40 (29.6; 21.9–37.3)	p = 0.607 ǂ
STDR (n; %; 95% CI)	10 (67%) (43.2–90.8)	25 (19.1%) (12.4–25.8)	17 (12.6; 7.0–18.2)	p = 0.021* ǂ
Proliferative DR (n; %; 95%CI)	1 (7%) (0–19.9)	10 (7.6%) (3.1–12.1)	3 (2.2; 0–4.7)	p = 0.054 ǂ

* = statistically significant

^†^Unpaired t-test

^ǂ^ Fisher’s exact

^ł^ Wilcoxon rank sum

^≠^ Χ^2^ test for trend

DR = diabetic retinopathy; STDR = Sight threatening diabetic retinopathy; HbA1c = glycated haemoglobin; NGSP = National Glycohemoglobin Standardization Program; HIV = Human immunodeficiency virus; sBP = systolic blood pressure.

**Table 2:** Baseline (2007) demographic, clinical and biochemical measurements for 135 subjects included in the 2007 study and seen again in 2012. Subjects categorised by grade of retinopathy at baseline: no DR, non-proliferative DR (LDES Level 20–50), and proliferative DR (LDES Level ≥60). 2 subjects ungradable at baseline and therefore not included.

Characteristic	No DR	Non-proliferative DR	Proliferative DR
**n**	93	37	3
**Age (yrs; med, IQR)**	52 (45–59)	54 (47–58)	46 (44–50)
**Type 1 diabetes**	12 (13%)	4 (11%)	2 (67%)
**Duration (yrs; med, IQR)**	2.8 (1.4–6.2)	6.2 (2.9–11.0)	8.5 (3.7–13.1)
**sBP (mmHg; med, IQR)**	130 (120–140)	135 (120–150)	170 (170–175)
**HbA1c (NGSP%; mean, SD)**	9.3 (2.8)	9.5 (2.2)	10.2 (1.4)
**HIV positive**	10 (10.8%)	5 (13.5%)	0
**Urine dipstick**	None 35; trace 28; 1+ 17; 2+ 5; 3+ 1; 4+ 0	None 8; trace 14; 1+ 11; 2+ 1; 3+ 2; 4+ 1.	None 0; trace 1; 1+ 1; 2+ 0; 3+ 1; 4+ 0.

DR = diabetic retinopathy; LDES = Liverpool diabetic eye study; sBP = systolic blood pressure; HbA1c = glycated haemoglobin; NGSP = National Glycohemoglobin Standardization Program; HIV = Human immunodeficiency virus.

Of the 135 subjects seen in the 2007 study and subsequently traced and assessed in 2012, 15 (11.1%) were HIV positive at baseline. By 2012 17 (12.6%) were reactive: 13 taking ART; 3 known HIV+ but not taking ART; and 1 new diagnosis (WHO stage 3) [22]. 21 subjects (15.6%) declined testing. In 2012 26 subjects (19.3%; 8 men, 18 women) were anaemic as defined above. The number of subjects who were hypertensive in 2007 and 2012 was 94 (69.6%) and 98 (72.6%), respectively. Only one subject received a course of scatter laser between 2007 and 2012. Of 117 subjects with type 2 diabetes, at baseline (2007) 22 (18.8%) were prescribed insulin (with or without oral hypoglycaemics), 88 (75.2%) were managed with oral hypoglycaemics and 7 (6.0%) were managed with diet alone. At the follow-up visit (2012) 40 (34.2%) were prescribed insulin, 70 (59.8%) were managed with oral hypoglycaemics and 7 (6.0%) were managed with diet alone.

**Fig 1:**
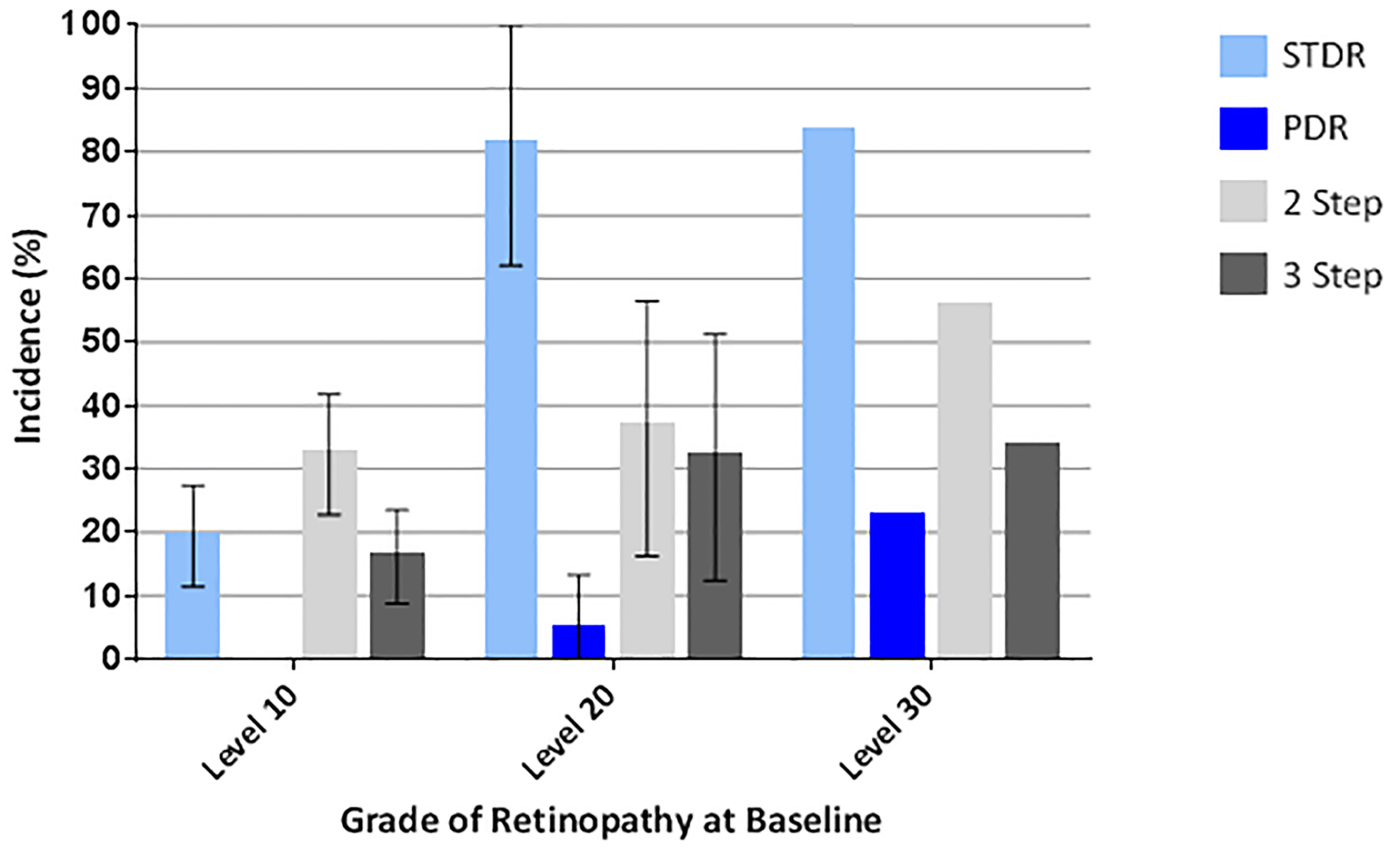
Five year incidence of progression to sight threatening diabetic retinopathy (STDR), proliferative diabetic retinopathy (PDR; level 60+), and of 2 (or more) step and 3 (or more) step progression on the LDES scale for subjects with diabetes and level 10 (n = 93), level 20 (n = 23) and level 30 (n = 9) retinopathy at baseline. Error bars indicate 95% CI.

Between baseline and 5 years prevalence of any retinopathy, STDR and PDR increased from 29.6% (95% CI 21.9–37.3) to 63.0% (54.8–71.1), 12.6% (7.0–18.2) to 38.5% (30.3–46.7) and from 2.2% (0–4.7) to 5.9% (1.9–9.9), respectively (n = 135). Two (or more) step progression (from baseline) was observed in 48 subjects (36.4%; 95% CI 28.2–44.6); three (or more) step progression in 30 subjects (22.7%; 15.6–29.9). Of 115 subjects without STDR at baseline (2007) 34 (29.6%; 21.3–37.9) had developed the condition at five years (Fig 1; S1–S4 Tables). Higher baseline HbA1c, longer duration of diabetes and lower haemoglobin were risk factors for 2 step progression in multivariate analysis (Table 3).

**Table 3:** Risk factors for association of progression of diabetic retinopathy by 2 or more steps on the Liverpool Diabetic Eye Study scale at 5 years in 132 subjects with diabetes. (Univariate and multivariate logistic regression; multivariate model populated using backwards stepwise selection).

	OR	95% CI	p value
**Univariate logistic regression**			
HbA1c (NGSP %)	1.37	1.13–1.66	0.001*
Duration (years)	1.05	0.99–1.12	0.096
Baseline grade of DR	1.51	1.05–2.17	0.026*
Type 1 diabetes	1.90	0.70–5.17	0.210
sBP (mmHg)	1.00	0.92–1.34	0.872
Haemoglobin (g/dL)	0.86	0.72–1.02	0.086
Log[urine ACR] (mg/mmol)	1.28	0.83–1.95	0.261
LDL cholesterol (mmol/L)	0.91	0.61–1.37	0.658
HDL cholesterol (mmol/L)	0.72	0.29–1.75	0.466
Triglycerides (mmol/L)	0.64	0.38–1.08	0.093
HIV positive	0.94	0.32–2.75	0.903
Age (years)	0.97	0.94–1.01	0.101
Sex (male)	0.70	0.32–1.54	0.378
**Multivariate logistic regression**			
HbA1c (NGSP %)	1.23	1.03–1.48	0.020*
Haemoglobin (g/dL)	0.77	0.62–0.98	0.033*
Duration (years)	1.08	0.99–1.16	0.060
Triglycerides (mmol/L)	0.66	0.35–1.24	0.195
Age (years)	0.97	0.92–1.01	0.094

* = statistically significant;

DR = diabetic retinopathy; HbA1c = glycated haemoglobin; NGSP = National Glycohemoglobin Standardization Program; sBP = systolic blood pressure; ACR = albumin creatinine ratio; LDL = low density lipoprotein; HDL = high density lipoprotein; HIV = Human immunodeficiency virus.

Over the follow-up period 25 subjects (18.8%) lost 5 or more ETDRS letters of which 7 subjects (5.3%) lost 15 or more letters. Of these 25 subjects 2 (1.5%) progressed to moderate visual impairment (50–59 letters) and 3 (2.3%) became ‘severely visually impaired or blind’ (<50 letters). The most common primary causes of visual loss for the 25 subjects who lost five or more letters were DR (36%) both DR and cataract (20%) and cataract alone (28%). Therefore in 56% of cases DR was the sole or equal contributing cause of visual loss. In univariate analysis loss of 5 or more ETDRS letters was associated with presence of STDR at follow up visit (OR 3.29, 95% CI 1.32–8.18, p = 0.010) but not age (OR 1.03, 0.99–1.07, p = 0.179) or duration of diabetes (OR 1.03, 0.96–1.10, p = 0.389).

**Fig 2:**
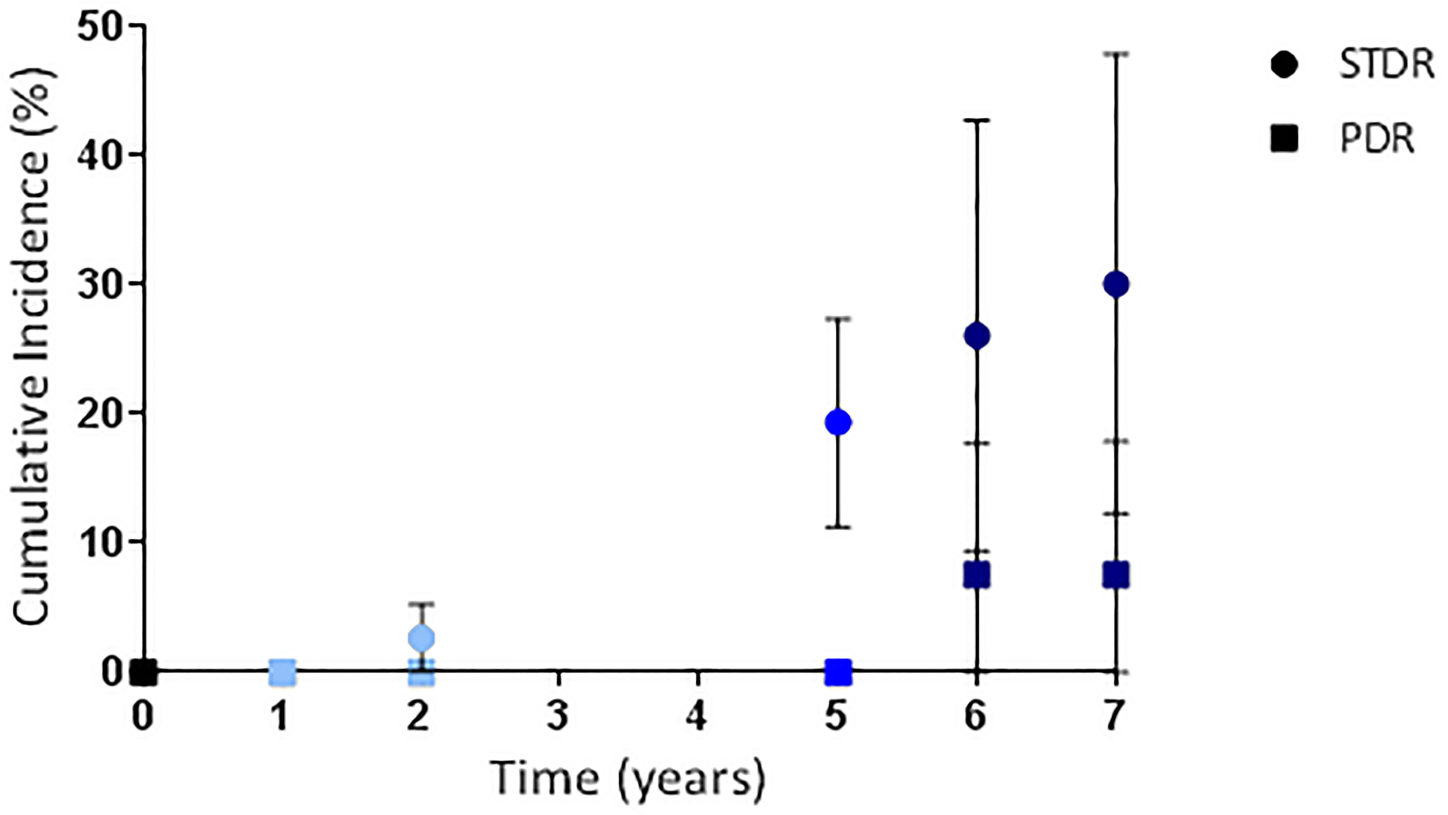
Composite graph showing incidence of sight threatening diabetic retinopathy (STDR) and proliferative diabetic retinopathy (PDR; level 60+) for subjects with diabetes and no diabetic retinopathy at baseline. Figures for 0, 1 and 2 years taken from our group’s 24 month cohort study performed between 2012 and 2014 and published elsewhere [14,15] (light blue symbols; n = 177); 5 years from subjects in the 2007 cohort traced and assessed in 2012 (mid blue symbols; n = 93); 6 and 7 years from subjects in the 2007 cohort who were recruited (systematic random sampling) into the 24 month cohort study in 2012 and therefore followed for a further 2 years (dark blue symbols; n = 27). Error bars indicate 95% CI.

Of the 135 subjects from the 2007 cohort 41 were recruited (by systematic random sampling from the QECH diabetes clinic) into our 24 month cohort study. Of the 41 subjects; 38 were seen at 12 months and 36 at 24 months providing 6 and 7 year longitudinal data (Table 4 and S5 Table). Of 34 subjects without STDR at baseline (2007) and seen at 7 years 12 (35.3%; 19.2–51.4) had developed the condition at 7 years. Fig 2 and S3 Fig combine data from our 24 month cohort study performed between 2012 and 2014 and published elsewhere [14,15], 5 year progression data detailed in this manuscript and 6 and 7 year data from the 41 subjects described above to give a composite graph of DR progression over 7 years.

**Table 4:** Life tables showing incidence at 5, 6 and 7 years of development of grades of retinopathy, sight threatening maculopathy, and sight-threatening diabetic retinopathy in the worse eye of 27 subjects with diabetes and no retinopathy (level 10) at baseline.

	**Any retinopathy**				**Level 30**				**Level 40**			
T	N	n	C. Inc.	95% CI	N	n	C. Inc.	95% CI	N	n	C. Inc.	95% CI
5	27	14	51.9	34.0–69.3	27	1	3.70	0–10.8	27	2	7.4	0–17.3
6	13	1	55.5	36.8–74.3	26	3	15.0	1.4–28.6	25	1	11.2	0–23.2
7	12	3	67.2	48.9–84.4	22	0	15.0	1.1–28.9	23	0	11.2	0–23.4
	**Level 60+**				**STDR**							
T	N	n	C. Inc.	95% CI	N	n	C. Inc.	95% CI				
5	27	0	0		27	5	18.5	3.9–33.2				
6	27	2	7.6	0–17.7	22	2	26.1	9.4–42.8				
7	24	0	7.6	0–17.9	19	1	30.1	12.3–47.9				

T = time from recruitment (years); N = number entering time interval; n = new cases diagnosed during year; C. inc. = cumulative incidence (%); CI = confidence interval; ST = sight threatening; STDR = sight threatening diabetic retinopathy.

## Discussion

We report the progression of grades of retinopathy and visual impairment over 5 years in a cohort of people with diabetes from Southern Malawi who had no access to laser treatment. Five year incidence of any DR in those without evidence of retinopathy at baseline was 48.4% (38.2–58.5). The five year incidence of STDR for those with level 10 and level 20 retinopathy at baseline was 19.4% (11.3–27.4) and 81.3% (62.1–100), respectively. The five year incidence of PDR for those with level 10, level 20, level 30 and level 40 retinopathy at baseline was 0%, 4.5%, 22% and 40%, respectively. Higher glycosylated haemoglobin (HbA1c) and lower haemoglobin level were risk factors for progression of retinopathy in multivariate analysis. Over the follow-up period 25 subjects (18.8%) lost 5 or more ETDRS letters of which 7 subjects (5.3%) lost 15 or more ETDRS letters. 2 subjects (1.5%) progressed to moderate visual impairment (50–59 letters) and 3 (2.3%) became ‘severely visually impaired or blind’ (<50 letters). In 56% of cases DR was the sole or equal contributing cause of visual loss.

High quality cohort studies of DR from Africa or Asia are scarce. In our group’s separate prospective cohort study performed in Malawi between 2012 and 2014, 2 year incidence of STDR for subjects with level 10 and level 20 retinopathy at baseline was 2.7% (95% CI 0.1–5.3) and 27.3% (16.4–38.2), respectively [15]. A subset of this cohort are taking part in further follow-up to investigate the efficacy of laser treatment for DR in this population. In Mauritius subjects examined in a population based study in 1992 [23] were re-examined for complications of diabetes in 1998 [24]. At 6 years incidence of DR and PDR in subjects with diabetes but no DR in the first study was 23.8% and 0.4%, respectively. The 5 year incidence of any DR in our study was higher (48.4%). For subjects with mild non-proliferative DR (equating to LDES level 20) and moderate non-proliferative DR (LDES L30 or L40) the incidence of PDR at 6 years was 5.2% and 29.4%, respectively. These figures are similar to the five year incidences of PDR in our study (level 20 4.5%; level 30 22%; level 40 40%). Compared to recent European studies progression to STDR from no DR (level 10) and background DR (level 20) was approximately 5 times higher (19.4% vs estimates between 3.9% [2,25] and 4.0% [26]) and 3 times higher (81.3% vs estimates between 26.8 [25] and 28.9 [2]), respectively. These disparities may be explained by differences in access to health services, standards of care for diabetes, presence of comorbidities and genetic factors. Comparisons between studies must be made with caution in view of different study designs and different follow-up rates. Of particular note mortality rates are likely to differ greatly between populations and are an important cause of data censoring.

DR progression at 5 years was associated with lower haemoglobin level. In our group’s prospective, 24 month cohort study lower haemoglobin was associated with presence of STDR at baseline (reported elsewhere [14]) but not with progression of DR [15]. An association between anaemia and presence of DR has been reported in Indian [27–29] and Chinese [30] cross-sectional studies. Potential confounders of this relationship are decreased erythropoietin production due to diabetic nephropathy and nutritional and socioeconomic status. Impaired oxygen delivery to retinal tissue is one possible mechanism to explain this association. No interventional studies have been conducted to test the effect of treatment of anaemia on the complications of diabetes. Both high iron level and iron supplementation have been associated with gestational diabetes [31,32] and therefore pose risks. In the majority of reports duration is a strong determinant of DR progression [3,4,6]. In this study, and in our group’s 24 mouth cohort study of DR in Southern Malawi [15], duration of diabetes was not associated with DR progression in multivariate logistic regression. Information bias may explain why no relationship was demonstrated. It is expected that time from onset of type 2 diabetes until diagnosis will be on average longer and more variable in low resource settings than in high resource settings.

To our knowledge, ours is the first study to report longitudinal visual acuity data in African subjects with diabetes. Procedures for assessing vision differed between the baseline and final assessments. ETDRS measurements yield better VA than Snellen measurements and differences are more marked in persons with low vision [33–35]. The effect of these differences in this study would be to reduce the degree of visual loss recorded. A potential bias is that subjects who became visually impaired may have been less likely to attend the follow-up visit in 2012. Visual impairment may increase the chance of mortality in a society where loss of vision entails loss of economic productivity. While laser treatment was not available to the vast majority of subjects in our study, other interventions including cataract surgery were obtainable. Together these factors may explain why a greater degree of visual impairment was not seen in this cohort study.

This clinic-based study has limitations. Barriers to attendance include lack of knowledge of available health services, transportation costs, and competing tasks such as planting and harvesting staple crops. Patients living in rural locations endure long journeys to clinic and may represent a selected sub-group of the rural diabetes population. Few subjects with diet controlled diabetes attend the QECH diabetes clinic [12,13] and therefore only a small number of these patients appeared in our study. Loss to follow-up is a source of bias in this study. Although relatively few deaths were confirmed, high mortality is likely to be an important cause of data censoring. As diabetes care improves in sub-Saharan Africa the prevalence of retinopathy may (paradoxically) go up due to case survival. The epidemic of diabetes in Sub-Saharan Africa necessitates provision of services. This study provides data which is essential in order to design and test locally appropriate and sustainable interventions for DR prevention, early detection and management in the region.

## Supporting information

S1 Fig Levels of retinopathy and maculopathy in the Liverpool Diabetic Eye Study. (DOCX)

S2 Fig Flow diagram for subjects in the Malawi Diabetic Retinopathy Study of DR progression at 5 years: enrolment in 2007; follow-up in 2012. (DOCX)

S3 Fig Composite graph showing incidence of sight threatening diabetic retinopathy (STDR) and proliferative diabetic retinopathy (PDR; level 60+) for subjects with diabetes and Level 20 retinopathy (and no STDR) at baseline. (DOCX)

S1 Table Five year incidence of development of grades of retinopathy, sight threatening diabetic retinopathy (STDR), and of progression by 2 (or more) and 3 (or more) steps on the LDES scale in the worse eye of 93 subjects with no retinopathy (level 10) at baseline. (DOCX)

S2 Table Five year incidence of development of grades of retinopathy, sight threatening diabetic retinopathy (STDR), and of progression by 2 (or more) and 3 (or more) steps on the LDES scale in the worse eye of 23 subjects with level 20 retinopathy at baseline. (DOCX)

S3 Table Five year incidence of development of grades of retinopathy, sight threatening diabetic retinopathy (STDR), and of progression by 2 (or more) and 3 (or more) steps on the LDES scale in the worse eye of 9 subjects with level 30 retinopathy at baseline. (DOCX)

S4 Table Five year incidence of development of proliferative DR (PDR), sight threatening maculopathy, and of progression by 2 (or more) and 3 (or more) steps on the LDES scale in the worse eye of 5 subjects with level 40 retinopathy at baseline. (DOCX)

S5 Table Life tables showing incidence at 5, 6 and 7 years of development of proliferative diabetic retinopathy (PDR; Level 60+), sight threatening maculopathy and sight-threatening diabetic retinopathy in the worse eye of 7 subjects with diabetes and level 20 retinopathy at baseline. (DOCX)
