# Supplementary material for: Incidence and progression of diabetic retinopathy in Sub-Saharan Africa: A five year cohort study
Source: PLoS One. 2017 Aug 2;12(8):e0181359. doi: 10.1371/journal.pone.0181359 (PMC5540405; doi:10.1371/journal.pone.0181359)
Supplement: S1 Fig — (DOCX) [file pone.0181359.s001.docx]

**S1 Figure** Levels of retinopathy and maculopathy in the Liverpool Diabetic Eye Study [16]

**Level Definition**

**Retinopathy**

1. No retinopathy

20 Haemorrhages or microaneurysms < ETDRS standard photograph 2A †

30 Haemorrhages or microaneurysms ≥ETDRS standard photograph 2A, and/or 1-6 cotton wool spots

40 Haemorrhages/ microaneurysms ≥ ETDRS standard photograph 2A

and/or ≥ 6 cotton wool spots

and/or 1 quadrant venous changes

and/or IRMA < ETDRS standard photograph 8A

50 IRMA ≥ ETDRS standard photograph 8A

and/or 2 or more quadrants venous changes

and/or pre-retinal haemorrhage in absence of proliferation

60 Fibrovascular proliferation

and/or proliferative retinopathy

70 Diabetic Retinopathy Study high risk characteristics

71 Tractional retinal detachment

72 No fundal view due to vitreous blood

90 Ungradeable due to any other reason e.g. media opacity

**Maculopathy**

0 No maculopathy

1 Questionable: < 50% certainty of presence of exudate

2 Exudate >1 disc diameter from fixation

3 Circinate ring of exudate within macula >1 disc area in size but not within 1 disc diameter of fixation

4 Exudates within 1 disc diameter of fixation

and/or presence of clinically significant macular oedema

8 Exudates due to other diseases e.g. vein occlusion, choroidal neovascularisation

90 Ungradeable

† Definition of any diabetic retinopathy: ≥ 1 haemorrhage or microaneurysm in either eye. Flame shaped haemorrhages associated with hypertension are discounted. IRMA = intraretinal microvascular abnormalities. ETDRS = Early Treatment Diabetic Retinopathy Study.
