# Supplementary material for: Incidence and progression of diabetic retinopathy in Sub-Saharan Africa: A five year cohort study
Source: PLoS One. 2017 Aug 2;12(8):e0181359. doi: 10.1371/journal.pone.0181359 (PMC5540405; doi:10.1371/journal.pone.0181359)
Supplement: S2 Fig — (DOCX) [file pone.0181359.s002.docx]

**S2 Figure** Flow diagram for subjects in the Malawi Diabetic Retinopathy Study of DR progression at 5 years: enrolment in 2007; follow-up in 2012.

**Enrolment 2007**

Not examined for retinopathy in original study (n=339)

Slit lamp fundus examination in original study (n=281)

Not traced (n=121)

Participants in original cross sectional study of complications of diabetes (n=620)

**Subject tracing 2012**

Confirmed dead (n=15)

Alive but not assessed (n=10)

• Traced but declined assessment (n=7)

• Moved away from Southern Malawi (n=3)

Patients assessed (n=135)

• Recruited to the 24 month cohort study (n=41)

• Traced by study team (n=94)

Anaemia
