# Supplementary material for: Incidence and progression of diabetic retinopathy in Sub-Saharan Africa: A five year cohort study
Source: PLoS One. 2017 Aug 2;12(8):e0181359. doi: 10.1371/journal.pone.0181359 (PMC5540405; doi:10.1371/journal.pone.0181359)
Supplement: S3 Fig — (DOCX) [file pone.0181359.s003.docx]

**S3 Figure** Composite graph showing incidence of sight threatening diabetic retinopathy (STDR) and proliferative diabetic retinopathy (PDR; level 60+) for subjects with diabetes and **Level 20 retinopathy** (and no STDR) at baseline. Figures for 0, 1 and 2 years taken from our group’s 24 month cohort study performed between 2012 and 2014 and published elsewhere [14,15] (light blue symbols; n=94); 5 years from subjects in the 2007 cohort traced and assessed in 2012 (mid blue symbols; n=23); 6 and 7 years from subjects in the 2007 cohort who were recruited (systematic random sampling) into the 24 month cohort study in 2012 and therefore followed for a further 2 years (dark blue symbols; n=7). Error bars indicate 95% CI.
