# Supplementary material for: Incidence and progression of diabetic retinopathy in Sub-Saharan Africa: A five year cohort study
Source: PLoS One. 2017 Aug 2;12(8):e0181359. doi: 10.1371/journal.pone.0181359 (PMC5540405; doi:10.1371/journal.pone.0181359)
Supplement: S1 Table — (DOCX) [file pone.0181359.s004.docx]

**S1 Table** Life table showing five year incidence of development of grades of retinopathy, sight threatening diabetic retinopathy (STDR), and of progression by 2 (or more) and 3 (or more) steps on the LDES scale in the worse eye of 93 subjects with **no retinopathy** (level 10) at baseline. n= number of subjects reaching endpoint.

| **Grade progression** | **Number entering time interval** | **n** | **Incidence %**  **(95% CI)** |
| --- | --- | --- | --- |
| 10 - 10 | 93 | 48 | 51.6 (41.5-61.8) |
| 10 - 20 | 93 | 30 | 32.3 (22.8-41.8) |
| 10 - 30 | 93 | 9 | 9.7 (3.7-15.7) |
| 10 - 40 | 93 | 6 | 6.5 (1.5-11.5) |
| 10 – 50+ | 93 | 0 | 0 |
| 10 - 20+ (any DR) | 93 | 45 | 48.4 (38.2-58.5) |
| 10-STDR | 93 | 18 | 19.4 (11.3-27.4) |
| 10-2+ step progression | 93 | 30 | 32.3 (22.8-41.8) |
| 10-3+ step progression | 93 | 15 | 16.1 (8.7-23.6) |
