# Supplementary material for: Incidence and progression of diabetic retinopathy in Sub-Saharan Africa: A five year cohort study
Source: PLoS One. 2017 Aug 2;12(8):e0181359. doi: 10.1371/journal.pone.0181359 (PMC5540405; doi:10.1371/journal.pone.0181359)
Supplement: S2 Table — (DOCX) [file pone.0181359.s005.docx]

**S2 Table** Five year incidence of development of grades of retinopathy, sight threatening diabetic retinopathy (STDR), and of progression by 2 (or more) and 3 (or more) steps on the LDES scale in the worse eye of 23 subjects with **level 20 retinopathy** at baseline. n =number of subjects reaching endpoint.

| **Grade progression** | **Number entering time interval** | **n** | **Incidence %**  **(95% CI)** |
| --- | --- | --- | --- |
| 20 - 10 | 22 | 1 | 4.5 (0-13.3) |
| 20 - 20 | 22 | 9 | 40.9 (20.4-61.5) |
| 20 - 30 | 22 | 5 | 22.7 (5.2-40.2) |
| 20 - 40 | 22 | 4 | 18.2 (2.1-34.3) |
| 20 - 50 | 22 | 2 | 9.1 (0-21.1) |
| 20 - 60+ | 22 | 1 | 4.5 (0-13.3) |
| 20 – STDR | 16 | 13 | 81.3 (62.1-100) |
| 20-2+ step progression | 22 | 8 | 36.4 (16.3-56.5) |
| 20-3+ step progression | 22 | 7 | 31.8 (12.4-51.3) |
