# Supplementary material for: Incidence and progression of diabetic retinopathy in Sub-Saharan Africa: A five year cohort study
Source: PLoS One. 2017 Aug 2;12(8):e0181359. doi: 10.1371/journal.pone.0181359 (PMC5540405; doi:10.1371/journal.pone.0181359)
Supplement: S3 Table — (DOCX) [file pone.0181359.s006.docx]

**S3 Table** Five year incidence of development of grades of retinopathy, sight threatening diabetic retinopathy (STDR), and of progression by 2 (or more) and 3 (or more) steps on the LDES scale in the worse eye of 9 subjects with level 30 retinopathy at baseline. n =number of subjects reaching endpoint.

| **Grade progression** | **Number entering time interval** | **n** | **Incidence %** |
| --- | --- | --- | --- |
| 30 - 30 | 9 | 3 | 33 |
| 30 - 40 | 9 | 4 | 44 |
| 30 - 50 | 9 | 0 | 0 |
| 30 - 60+ | 9 | 2 | 22 |
| 30 – STDR | 6 | 5 | 83 |
| 30-2+ step progression | 9 | 5 | 56 |
| 30-3+ step progression | 9 | 3 | 33 |
