# Supplementary material for: Incidence and progression of diabetic retinopathy in Sub-Saharan Africa: A five year cohort study
Source: PLoS One. 2017 Aug 2;12(8):e0181359. doi: 10.1371/journal.pone.0181359 (PMC5540405; doi:10.1371/journal.pone.0181359)
Supplement: S4 Table — (DOCX) [file pone.0181359.s007.docx]

**S4 Table** Five year incidence of development of proliferative DR (PDR), sight threatening maculopathy, and of progression by 2 (or more) and 3 (or more) steps on the LDES scale in the worse eye of 5 subjects with level 40 retinopathy at baseline. n =number of subjects reaching endpoint.

| **Grade progression** | **Number entering time interval** | **n** | **Incidence %** |
| --- | --- | --- | --- |
| 40 - 60+ (PDR) | 5 | 2 | 40 |
| 40 - ST maculopathy | 5 | 4 | 80 |
| 40-2+ step progression | 5 | 2 | 40 |
| 40-3+ step progression | 5 | 2 | 40 |

n =number of subjects reaching endpoint
