# Supplementary material for: Incidence and progression of diabetic retinopathy in Sub-Saharan Africa: A five year cohort study
Source: PLoS One. 2017 Aug 2;12(8):e0181359. doi: 10.1371/journal.pone.0181359 (PMC5540405; doi:10.1371/journal.pone.0181359)
Supplement: S5 Table — (DOCX) [file pone.0181359.s008.docx]

**S5 Table** Life tables showing incidence at 5, 6 and 7 years of development of proliferative diabetic retinopathy (PDR; Level 60+), sight threatening maculopathy and sight-threatening diabetic retinopathy in the worse eye of 7 subjects with diabetes and level 20 retinopathy at baseline.

|  | **PDR** | | | **ST maculopathy‡** | | | **STDR‡** | | |
| --- | --- | --- | --- | --- | --- | --- | --- | --- | --- |
| T | N | n | C. Inc. | N | n | C. Inc. | N | n | C. Inc. |
| 5 | 7 | 1 | 14.3 | 6 | 4 | 66.7 | 6 | 4 | 66.7 |
| 6 | 6 | 1 | 28.6 | 2 | 1 | 83.3 | 2 | 1 | 83.3 |
| 7 | 5 | 1 | 42.9 | 1 | 1 | 100 | 1 | 1 | 100 |

T = time from recruitment (years); N = number entering time interval; n = new cases diagnosed during year; C. inc. = cumulative incidence (%); CI = confidence interval; ST = sight threatening; STDR = sight threatening diabetic retinopathy. ‡ - those with sight threatening maculopathy/STDR at baseline omitted from analysis
